# Single-particle tracking of polymer aggregates inside disordered porous media†

**DOI:** 10.1039/d4na00873a

**Published:** 2025-01-08

**Authors:** Yusaku Abe, Naoki Tomioka, Yu Matsuda

**Affiliations:** a Department of Modern Mechanical Engineering, Waseda University 3-4-1 Ookubo Shinhuku-ku Tokyo Japan ya-jupiter0309@toki.waseda.jp y.matsuda@waseda.jp

## Abstract

The diffusion motions of individual polymer aggregates in disordered porous media were visualized using the single-particle tracking (SPT) method because the motions inside porous media play important roles in various fields of science and engineering. In the aggregates diffused on the surfaces of pores, continuous adsorption and desorption processes were observed. The relationship between the size of the aggregates and pore size was analysed based on diffusion coefficients, moment scaling spectrum (MSS) slope analysis, and diffusion anisotropy analysis. The obtained diffusion coefficients were different for different aggregates and pore sizes. The MSS slope analysis indicated that more than 85% of the aggregates showed confined diffusion for all the conditions investigated. The diffusion anisotropy analysis suggested that the diffusion of the aggregates exhibited anisotropic behaviour. The interactions between the aggregates and the pores were complex, causing the aggregates to exhibit motions distinct from those associated with surface diffusion on smooth surfaces.

## Abstract 2

The diffusion motions of individual polymer aggregates in disordered porous media were visualized and analysed using the single-particle tracking (SPT) method.

## Graphical abstract

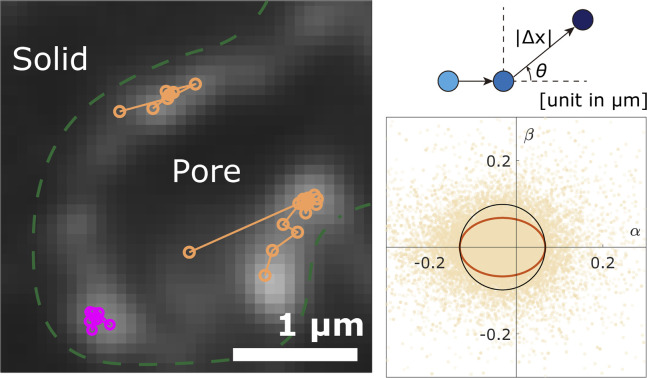

## Introduction

Porous media have been widely used in industries because of their unique properties, which are caused by their geometrical and surface interactions.^1^ For example, the porous media are used as catalysts,^2^ adsorbents,^3,4^ and filters.^5^ Since polymers tend to aggregate in solutions,^6–8^ the motion of polymers inside porous media depends not only on the properties of single polymer chains but also on polymer aggregates.^9^ Therefore, an understanding of the aggregate motions inside porous media is important for the designing of desired polymers and porous media.

The polymer aggregates diffuse into porous media *via* interactions with the pores. The interactions are assumed to vary with the aggregate size, pore size, and pore geometry. Previously, studies on the motion of nanoparticles inside the porous media were conducted, which shed light on the properties of their motion.^10–12^ However, further investigation into the behavior of polymer aggregates is required, as their frequently changing conformations significantly affect their motion properties. Sugar *et al.*^13^ revealed that the motion of the aggregates inside straight flow channels is affected by the size of the aggregates and width of the flow channels from the fluorescence imaging results of the flowing fluorescent polymers and their aggregates. Even though the interaction between the simple geometry of the channel and the aggregates induces complex behaviours, it has led to an increased interest in the behaviour of polymer aggregates in disordered pores with more complex geometries.

In our previous study, we have found that the heterogeneous structure of the disordered porous media is the only reason for the anisotropic diffusion motion of nanoparticles inside disordered porous media.^10^ Miyagawa *et al.*^14^ investigated the polymer behaviour inside a single mesoporous silica particle by measuring the distribution of the fluorescence intensity from polymers and revealed that the motion of polymers depends on the pore size. Skaug *et al.*^15^ adopted a single-molecule tracking method to visualize the surface diffusion of polymers on a substrate. However, these studies have not measured and analysed the motion of the individual polymer aggregates inside disordered porous media. However, visualization of the motion of the individual polymer aggregates is needed to reveal the features of the diffusion of aggregates.

In the present study, we adopted a single-particle tracking (SPT) method^16–18^ to investigate the polymer aggregate behaviour inside a monolithic silica column.^19^ The SPT method is a powerful tool for investigating the motion of individual probes because it directly records the motion using a fluorescence microscope. Recently, Tam *et al.*,^20^ have investigated the diffusion of nanoparticles in human tissues using the SPT method, and Xue *et al.*,^21^ revealed the hopping motion of nanoparticles in polymer solutions. Based on the trajectory data obtained by the SPT method, we can characterize the behaviour of the individual aggregates, such as diffusion coefficient, diffusion motion, and diffusion anisotropy. The monolithic silica columns used in this study had unimodal distribution of pore size. The diffusion motion of the polymer aggregates inside columns may be complex because the orientation, the pore size, and the shape inside columns are randomly arranged. In fact, our previous study^10^ has revealed that the nanoparticle motion inside the monolithic column is heterogeneous. We investigated the effects of the pore size and aggregation size on the motion of the aggregate. The suppression of the diffusion coefficients and the modes were investigated. The diffusion anisotropy was analysed by considering the displacement probability distributions (DPDs) and the relationship between the angle and the displacement obtained from three consecutive steps of trajectory.

## Materials and methods

### Sample preparation

**Fig. 1:**
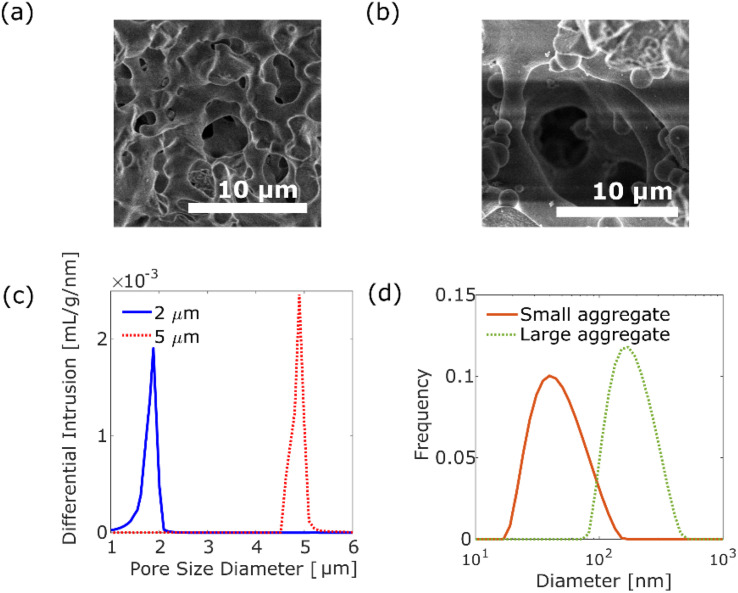
SEM images of silica monolithic columns with pore size distribution peaks at 2 μm (a) and 5 μm (b). (c) Pore size distributions of silica monolithic columns with pore size: the peak at 2 μm (solid blue line) and 5 μm (dashed red line). (d) Histogram of DLS of small aggregates with a diameter distribution peak at 40 nm (solid orange line) and large aggregates with a diameter distribution peak at 171 nm (dashed green line).

We investigated the diffusion motion of polymer aggregates in silica monolithic columns (Ex-Pure, Kyoto Monotech Co. Ltd., Japan) having disordered pore networks, where the silica monolithic columns were used as received. The silica monolithic column was originally designed for chromatography, and has been cleaned by the manufacturer. We examined that there was no fluorescence contamination inside the silica monolithic column using a fluorescence microscope. Fig. 1a and b show the scanning electron microscopic (SEM) images of the columns with pore size distribution peaks at 2 μm and 5 μm, respectively. As shown in Fig. 1a and b, the columns have disordered and continuous pores formed by polymerisation-induced phase separation.^19^ The pore size distributions of the columns were measured using a mercury porosimeter (AutoPore IV 9500, Shimadzu Co. Ltd., Japan), as shown in Fig. 1c. The columns with small (2 μm) and large (5 μm) pores have 1.0–2.1 μm and 4.5–5.2 μm pore sizes, respectively. The tortuosity factors of the columns were 1.58 and 1.51 for the columns with small (2 μm) and large (5 μm) pores, respectively. The porosity factors were 56.7% and 49.5% for the columns with small pores and large pores, respectively.

A luminescent poly(*p*-phenylenevinylene) (PPV) copolymer (Livilux®, PDY-132, Sigma-Aldrich, USA) was used as the probe polymer aggregate. We investigated the dependence of the size of polymer aggregates on diffusion motion. Two different sizes of aggregates were prepared from the 8.7 × 10^−3^ wt% PPV copolymer stock solution in toluene (99.5% pure, Fujifilm Wako Pure Chemical Corporation, Japan): one was prepared by passing the stock solution through a 0.2 μm syringe filter (Membrane Solutions Co. Ltd., USA). The size of the obtained aggregates was measured as 20–150 nm, centred at 40 nm (see Fig. 1d), using a dynamic light scattering (DLS) analyser (nanoPartica SZ-100V2, HORIBA Co. Ltd., Japan). The other aggregate was prepared by a reprecipitation method. The stock solution (2.5 mL) was added to 1 mL of methanol (99.8% pure, Fujifilm Wako Pure Chemical Corporation, Japan). After filtration using a 5 μm syringe filter (Membrane Solutions Co. Ltd., USA), the aggregates of 80–450 nm, centred at 171 nm, were obtained by evaporation of the solvent (see Fig. 1d). As shown in Fig. S2,† the absorption spectra and emission spectra for two different size aggregates are similar.

### SPT measurements

We measured the aggregate motions in the column using the same SPT measurement system used in our previous studies.^10,22,23^ Herein, we provide a brief explanation of the measurement. The SPT measurements were conducted at room temperature of 25 °C. The monolithic silica column was placed in a custom-made glass cell (diameter: 2.9 mm, depth: 1.4 mm) made on a cover glass (Thickness No. 1, Matsunami Glass Co. Ltd., Japan), and a dilute solution of the aggregates was poured into it. The fluorescence emitted from the aggregate was measured using an inverted fluorescence microscope (IX-73, Olympus Co. Ltd., Japan), a confocal scanner unit (CSU-X1, Yokogawa Electric Co. Ltd., Japan), and an oil-immersion objective lens of 100×, NA = 1.45, and WD = 0.13 mm (UPLXAPO100XO, Olympus Co. Ltd., Japan). The aggregates were photo-excited using a solid-state laser at an emission wavelength of 488 nm (OBIS488LS, Coherent CO. Ltd., USA). The output power of the laser was set at 135 mW. The fluorescence filtered by an optical bandpass filter (FF01-565/133-25, Semrock, USA) was captured using an electron-multiplying charge-coupled device (EMCCD) camera (C9100-23B, ImagEM X2, Hamamatsu Photonics Co. Ltd., Japan) at 50 frames per second (fps) (exposure time: 20 ms). Under this condition, the one pixel of the obtained image corresponded to an actual length of 0.08 μm. We obtained images in several cross sections of 20 to 30 μm from the glass surface by changing the focal plane using a piezo actuator (P-725K, Physik Instrumente GmbH & Co. KG, Germany). We conducted the SPT measurements under the following three combinations of conditions for the aggregates and pore sizes: (a) the aggregations with a diameter of *d* in the column with 126*d* pores, (b) the aggregations with a diameter of *d* in the column with 8*d* pores, and (c) the aggregations with a diameter of 0.2*d* in the column with 8*d* pores, where *d* is the diameter of the aggregates of 171 nm. Ten measurements were conducted for each condition, and 250 images were acquired for each measurement.

### Analysis of trajectories

Time-series images of the aggregate motion were analysed using ImageJ Fiji^24^ and PartcileTracker.^25–27^ Each aggregate trajectory was extracted using ParticleTracker with the appropriate parameters. For images of small aggregates, the parameters were set as radius = 5 px, cutoff = 0.001, and per/abs = 0.15. For images of large aggregates, parameters were set as radius = 6 px, cutoff = 0.001, and per/abs = 0.05. The position vector of an aggregate at time step *t* is represented by *X*(*t*), where *t* (= 0, 1, 2, …, *T*) represents the time step and *T* represents the total time steps of the trajectory. For the two-dimensional trajectory, the relationship between the ensemble average of the *ν*th moment of displacement and the lag time *τ* is expressed as follows:

1 〈‖*x*(*t* + *τ*) − *x*(*t*)‖^*ν*^〉 = 4*D*_*ν*_*τ*^*γ*^,

where *D*_*ν*_ is the generalized diffusion coefficient, *γ* is the scaling coefficient, 〈·〉 represents the ensembled average, and ‖·‖ represents the Euclidean norm. When *ν* = 2 and *γ* = 1, eqn (1) becomes the relationship between well-known mean squared displacement (MSD) and the regular diffusion coefficient *D*_*ν*=2_. Hereafter, we denote *D* = *D*_*ν*=2_ to simplify the notation. The moment scaling spectrum (MSS),^25,28^ the slope of which *S*_MSS_ is an indicator of diffusion modes of each aggregate, was calculated using eqn (1). For the Brownian diffusion, *S*_MSS_ was calculated as 0.5. In contrast, *S*_MSS_ was calculated as 0 < *S*_MSS_ < 0.5 for the confined diffusion. For super diffusion, *S*_MSS_ was calculated as 0.5 < *S*_MSS_ < 1. In the analysis, trajectories with fewer than 6 steps were not considered because short trajectories provided less reliable results. It is expected that adsorbed aggregates tend to be 0 < *S*_MSS_ < 0.5 and desorbed aggregates tend to be 0.5 < *S*_MSS_ < 1.

For a more detailed analysis, three consecutive steps of trajectories^29^ were investigated to extract the diffusion anisotropy. When three consecutive positions of **x**(*t* − 1), **x**(*t*), and **x**(*t* + 1) are considered, the displacement between later two positions **x**(*t* + 1) − **x**(*t*) and the angle *θ* between the displacement vectors **x**(*t*) − **x**(*t* − 1) and **x**(*t* + 1) − **x**(*t*) are plotted as a scatter diagram. We introduced *α* − *β* space to analyse the anisotropy. The position of the aggregates in the *α* − *β* space is defined as follows:2
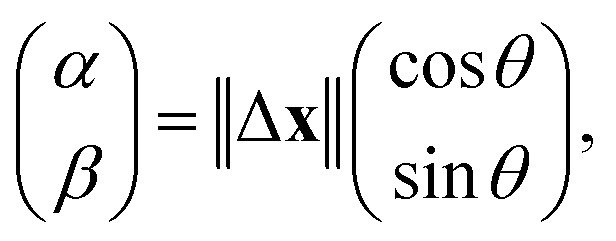
where ‖Δ**x**‖ = ‖**x**(*t*) − **x**(*t* − 1)‖. We calculated the average of *α* components and *β* components of the displacement and represented them as the radii of gyrations. By comparison with the radii of gyrations, we organized the dependence of the diffusion anisotropy on the porous structure and the size of the aggregates. The details of the explanation are provided in our previous studies.^10^

## Results and discussion

### Direct visualization of individual polymer aggregates

**Fig. 2:**
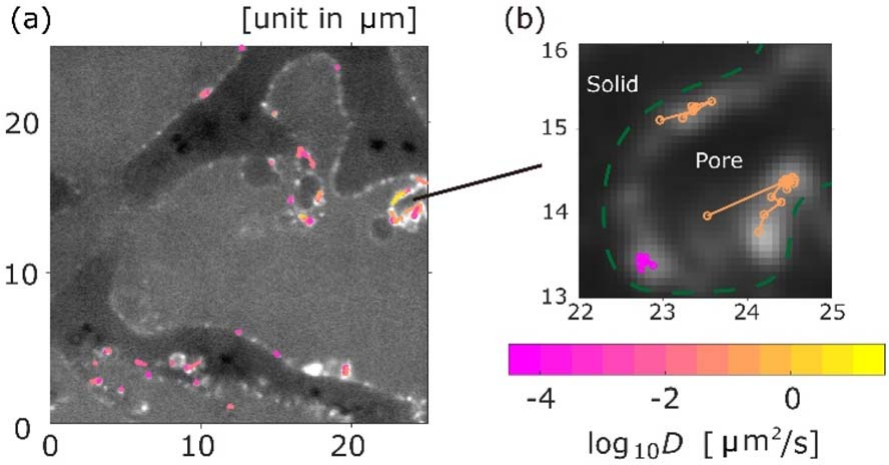
Typical image of the measured trajectories, where the size of aggregates is 171.3 nm and the pore size is 5 μm. Each trajectory is coloured by its diffusion coefficient *D*. (a) Trajectory distribution in area of 25 μm × 25 μm. (b) Trajectories with adsorption and desorption. Pore walls are highlighted with dashed green lines in (b).

The trajectories of the aggregates of 171 nm (*d*) coloured according to their diffusion coefficients are shown in Fig. 2, where a background image of the monolithic column with a pore size of 5 μm (29*d*) is a time-averaged measurement image obtained by SPT. As shown in Fig. 2a, most trajectories were located on the surface of pores. The diffusion coefficients were different for each trajectory. Fig. 2b is a magnified image. As shown in Fig. 2b, some aggregates were adsorbed in a certain area on the surface of the pore, while other aggregates diffused along the surface. Moreover, desorption from the surface was measured, as illustrated in Fig. 2b.

### Diffusion coefficient and MSS slope analysis

**Fig. 3:**
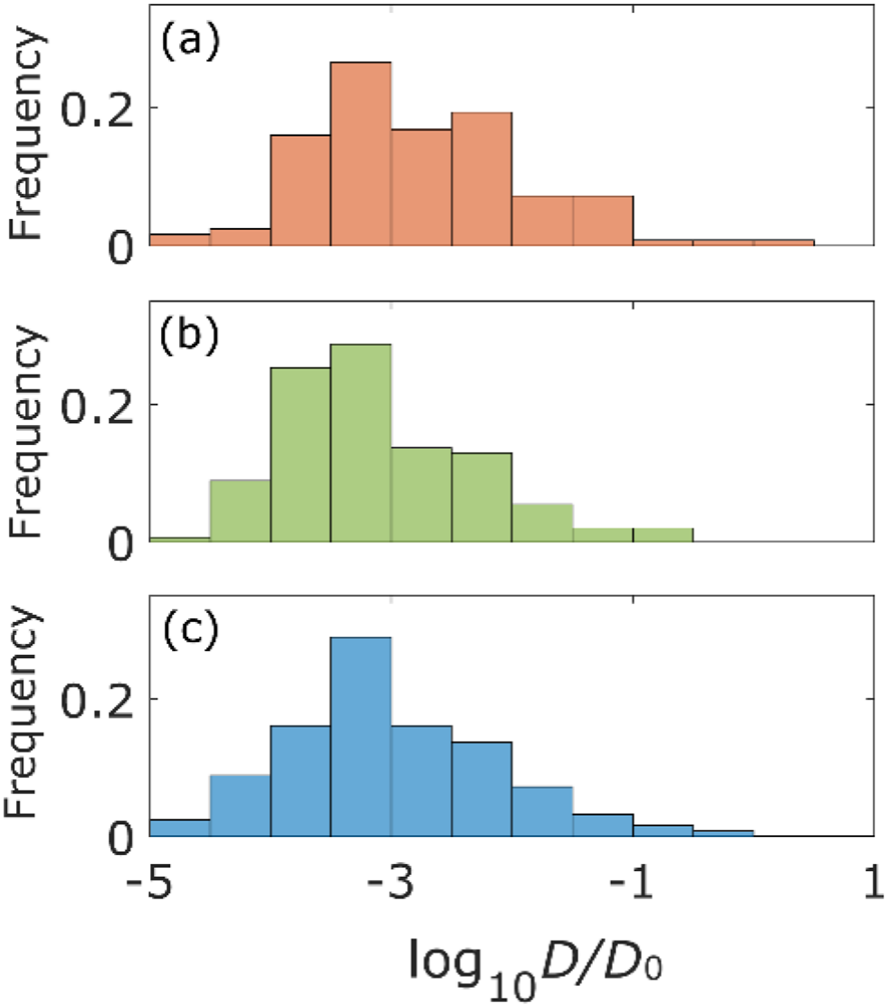
Histograms of the diffusion coefficient for the aggregates with diameter *d* for the columns with pore size of 126*d* (a) and 8*d* (b) and the aggregates with diameter 0.2*d* for the column with pore size 8*d* (c). Diffusion coefficients of (a) and (b) are normalized by *D*_0_ = 4.7 μm^2^ s^−1^ and that of (c) is normalized by *D*_0_ = 18.5 μm^2^ s^−1^.

The statistical properties of the motion of the aggregates were investigated. Fig. 3 shows the histograms of diffusion coefficients. The experimental conditions of Fig. 3a and b are the aggregates of diameter 171 nm (*d*) inside the column of pore size 126*d* and 8*d*, respectively. The experimental condition of Fig. 3c is the aggregate of diameter 0.2*d* inside the column of pore size 8*d*. The numbers of trajectories analysed in Fig. 3a–c are 1263, 1795, and 605, respectively. The diffusion coefficients were normalized by their bulk diffusion coefficients of 4.7 μm^2^ s^−1^ for the aggregates of diameter *d* and 18.5 μm^2^ s^−1^ for aggregates of diameter 0.2*d*, which were measured using a DLS analyser. Compared with the bulk diffusion coefficients, the average diffusion coefficients of these measurements were 6.7% (Fig. 3a), 3.4% (Fig. 3b), and 1.1% (Fig. 3c). Comparing the results displayed in Fig. 3a and b, it was observed that the diffusion motions in small pores were highly suppressed. In Fig. 3b and c, the larger the size of aggregates, the fewer the aggregates with large diffusion coefficients.

**Fig. 4:**
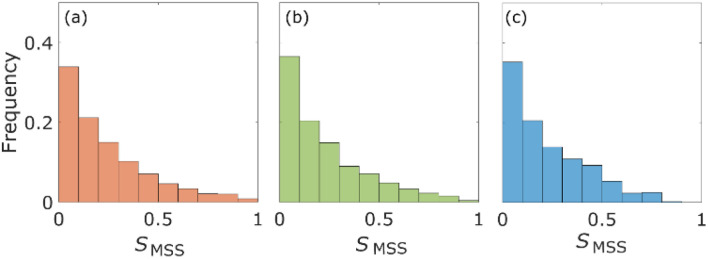
Histograms of the MSS slope *S*_MMS_ for the aggregates with diameter d for the columns with pore size 126*d* (a) and 8*d* (b), and the aggregates with diameter 0.2*d* for the column with pore size 8*d* (c).

The percentages of the aggregates for which 0 < *S*_MSS_ < 0.5 are 87.3 (Fig. 4a), 87.6 (Fig. 4b), and 89.8 (Fig. 4c), where *S*_MSS_ is the MSS slope. These results indicate that the diffusion motion of most aggregates is confined. Specifically, in conjunction with the visualization results shown in Fig. 2, most of the aggregates exhibit diffusion along the surface of the pore. Simon *et al.*^30^ suggested the idea of the confinement factor, which can characterize the sub-diffusive probes using machine learning methods. As shown in Fig. S3,† the diffusion mode of most aggregates with more than 20 step lengths was confined diffusion, similar to the result of the MSS slope analysis.

From Fig. 3 and 4, the diffusion coefficients decreased, and more aggregates were confined as the pore size decreased because the small pores on the surface of the columns would cause adsorption of the aggregates. Diffusion coefficients also decrease as the size of the aggregates increases due to the Stokes–Einstein law.^31^

#### Diffusion anisotropy analysis

**Fig. 5:**
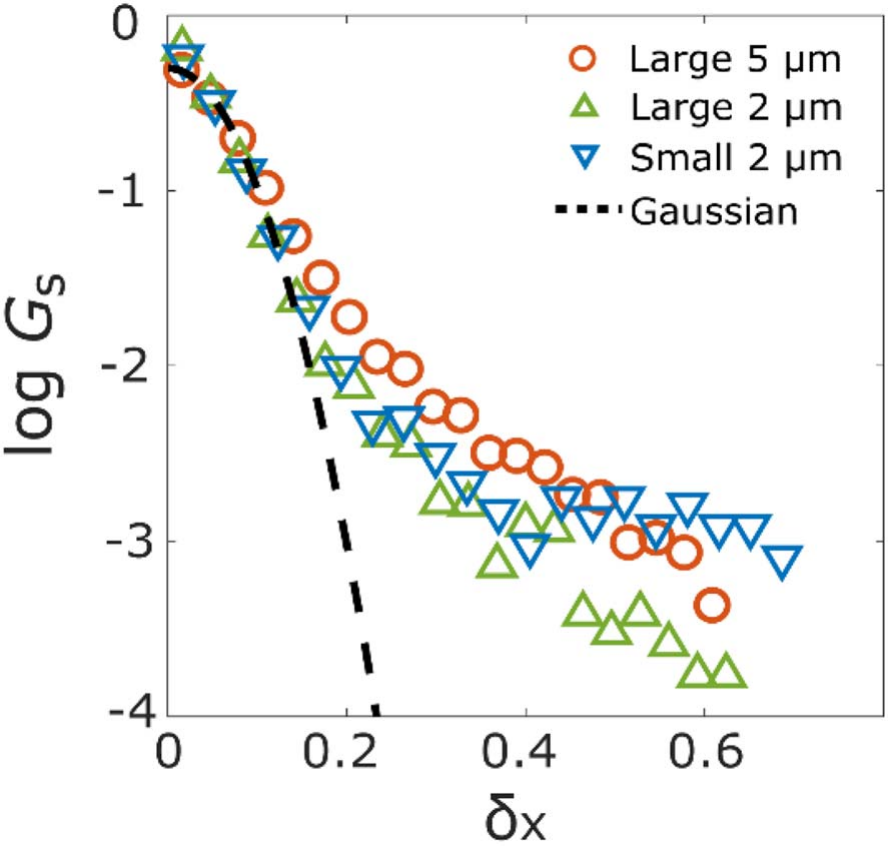
Displacement probability distribution (DPD) of the aggregates with diameter*d* for the columns with pore size 126*d* (orange circle) and 8*d* (green triangle), and the aggregates with diameter 0.2*d* for the column with pore size 8*d* (blue inverted triangle).

The displacement of the aggregates along the horizontal axis for each consecutive time step *δ*_*x*_ also reflects the underlying motion. It is well known that the displacement probability distribution (DPD) follows a Gaussian distribution in Fickian diffusion, while tails of DPD are not described by Gaussian distribution but exponential in anomalous diffusion.^32–35^ DPDs of the three experimental conditions are shown in Fig. 5, where *G*_S_ is the frequency of *δ*_*x*_ and the black dotted line shows a Gaussian distribution. Comparing the two DPDs with different pore sizes, the probability of a large displacement with the large pore size is higher than that with the small pore size. Furthermore, compared with two DPDs of different sizes of aggregates, the distributions for *δ*_*x*_ < 0.2 μm were similar. However, the DPD of small aggregates is higher than the DPD of large aggregates at *δ*_*x*_ > 0.2 μm. Additionally, we conducted the KS-test for revealing that the DPDs did not obey the Gaussian distribution.^36^ For the three experimental conditions, the results of the KS-test were rejected from the Gaussian distribution, as shown in Fig. S4.† These results indicate that the diffusion of the aggregates inside the disordered porous media is different from the Brownian diffusion.

**Fig. 6:**
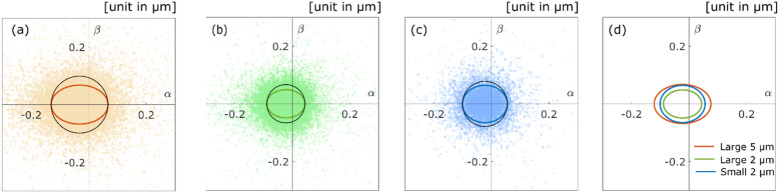
Scatter diagrams for the diffusion anisotropy of the aggregates with diameter *d* for the columns with pore size 126*d* (orange) (a) and 8*d* (green) (b), and the aggregates with diameter 0.2*d* for the column with pore size 8*d* (blue) (c). The coloured lines of ellipses are the radii of gyrations, and the black solid lines are the circles for which radii are equal to the radius of gyrations of the *α* axis. (d) Coloured lines of ellipses in (a)–(c) are overlayed.

The aggregates diffusing along the surface of the pore show high anisotropy because these aggregates move in a specific direction; namely, along the surface structure. We introduced the *α* − *β* space and calculated from the position of real space to the position of *α* − *β* space for the aggregate trajectory, according to the proposed method in a previous study.^29^ As shown in Fig. 6a–c, the aggregate positions of each time step in the *α* − *β* space are plotted for each experimental condition. We also calculated the radii of gyrations^29,37^ for the scatter plot to evaluate the diffusion anisotropy. The coloured solid lines in Fig. 6a–c are the radius of gyrations of each experimental condition. The black solid lines in Fig. 6a–c are the circles for which the radii are equal to the radius of gyration of the *α* axis. The ratios of the radius of the *α* axis *versus* the radius of the *β* axis/direction are 0.68 (Fig. 6a), 0.74 (Fig. 6b), and 0.83 (Fig. 6c). The result revealed that the polymer aggregates diffuse along the surface of the pore because these ratios are below 1 under all experimental conditions. Fig. 6d shows the overlaid plot of the radii of gyrations in Fig. 6a–c. Compared with the three radii, it is confirmed that displacements decrease as the size of the aggregate decreases, and the pore size increases. As shown in Fig. 6a and b, the diffusion anisotropy decreases as the pore size decreases. This result indicates that the aggregates tend to diffuse inside a small cavity or ledge on the surface when they were large relative to the size of the aggregates. The diffusion anisotropy also decreases as the size of the aggregates decreases. There are possible reasons as follows: first, the decrease in intermolecular forces due to decrease in the size of the aggregates trigger the desorption of the aggregates.^38^ Second, the roughness of the surface increases relatively as the size of the aggregates decreases.^39^ Finally, the adhesion force between the aggregates and the surface decreases due to changes in polymer conformation, and the conformation triggers the desorption of the aggregates.^15^

## Conclusions

We investigated the diffusion of polymer aggregates inside silica monolithic columns by the SPT method. By visualization of each trajectory obtained from the SPT method, it was revealed that the trajectories of each polymer aggregate located on the surface of pores. Statistical analysis of trajectories clarified that the distributions of diffusion coefficients are different for each experimental condition. Diffusion coefficients increase as the size of the aggregates decreases, and the pore size increases. DPD also suggests that there is a difference in aggregate behaviour. Diffusion anisotropy described the probability of aggregates moving along the surface of the pore. We found that the changes in the adsorption property of aggregates depend on the changes in the size of the aggregates and pore size. This study reveals that polymer aggregates diffuse heterogeneously inside disordered porous media. The results of this study indicate that the interactions between the aggregates and the pores differ depending on the size of the aggregates, the pore size, and the position of the aggregates on the surface.

## Data availability

The data supporting this article have been included in the manuscript or ESI.† The code of “ParticleTracker” used for extracting aggregates from images can be found at: https://sbalzarini-lab.org/?q=downloads/imageJ. The version of the code employed for this study is Version Nov 2016.

## Author contributions

Yusaku Abe: conceptualization, data curation, funding acquisition, formal analysis, investigation, methodology, software, validation, visualization, writing – original draft, and writing – review & editing. Naoki Tomioka: data curation, investigation, methodology, and software. Yu Matsuda: conceptualization, data curation, funding acquisition, investigation, methodology, project administration, supervision, and writing – review & editing.

## Conflicts of interest

There are no conflicts to declare.

## Supplementary Material

NA-OLF-D4NA00873A-s001
