# Supplementary material for: Single-particle tracking of polymer aggregates inside disordered porous media
Source: Nanoscale Adv. 2025 Jan 8;7(3):728–34. doi: 10.1039/d4na00873a (PMC11708829; doi:10.1039/d4na00873a)
Supplement: NA-007-D4NA00873A-s001 [file NA-007-D4NA00873A-s001.pdf]

Supplementary Information (SI) for Nanoscale Advances.
This journal is © The Royal Society of Chemistry 2024

Supplementary

Single particle tracking of polymer aggregates inside disordered porous media

Yusaku Abe[1,] *, Naoki Tomioka[1], Yu Matsuda[1,] *

1. Department of Modern Mechanical Engineering, Waseda University, 3-4-1 Ookubo, Shinjuku-ku, Tokyo, 169-8555, Japan

* corresponding author: Yusaku Abe, and Yu Matsuda

Email: ya-jupiter0309@toki.waseda.jp, y.matsuda@waseda.jp

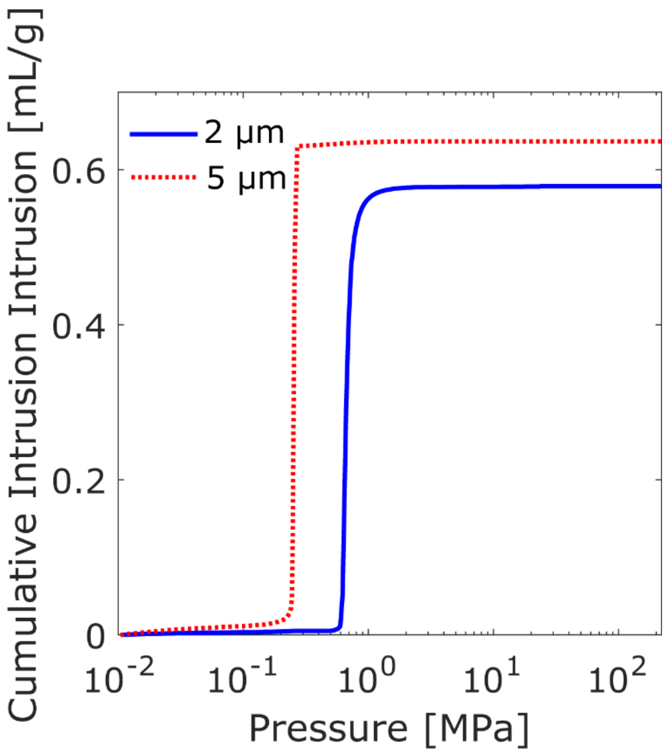

Fig. S1 Cumulative intrusion vs pressure plots of silica monolithic columns with pore size: the peak at 2 μm (solid blue line) and 5 μm (dashed red line).

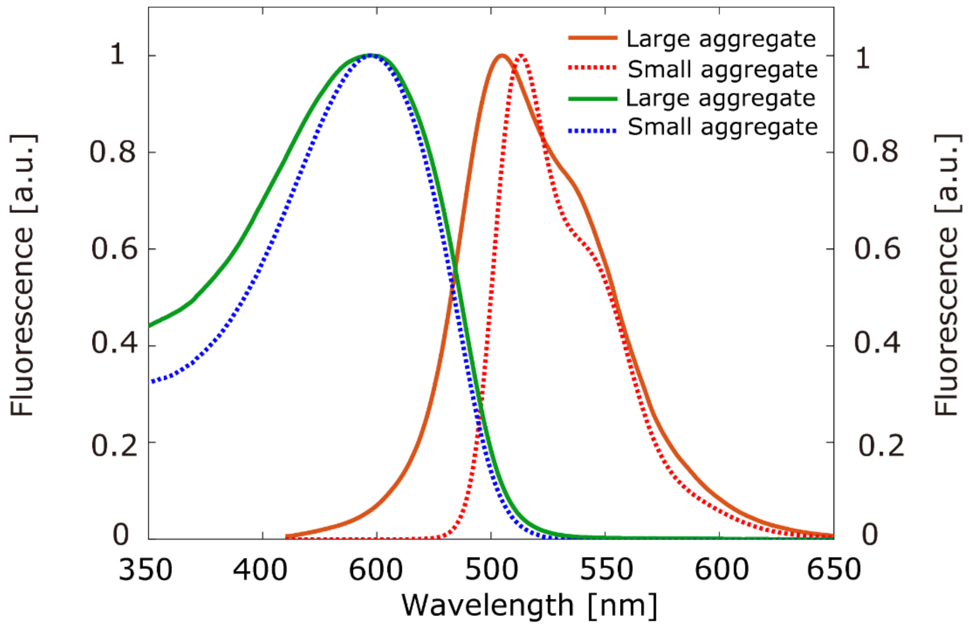

Fig. S2. Absorption spectra of large aggregates (solid green line) and the small aggregates (dashed blue line) and emission spectra of large aggregates (solid orange line) and small aggregates (dashed red line).

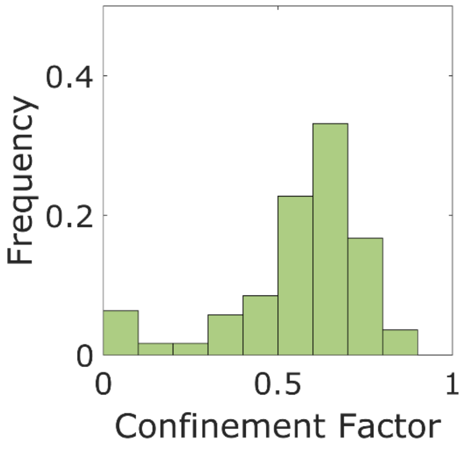

Fig. S3. Histogram of confinement factor, where the aggregates with diameter *d* for the column with pore size 8*d*. Details of the explanation of the confinement factor are provided by Simon, et al., eLife., 99347.1, 2024. https://doi.org/10.7554/eLife.99347.1

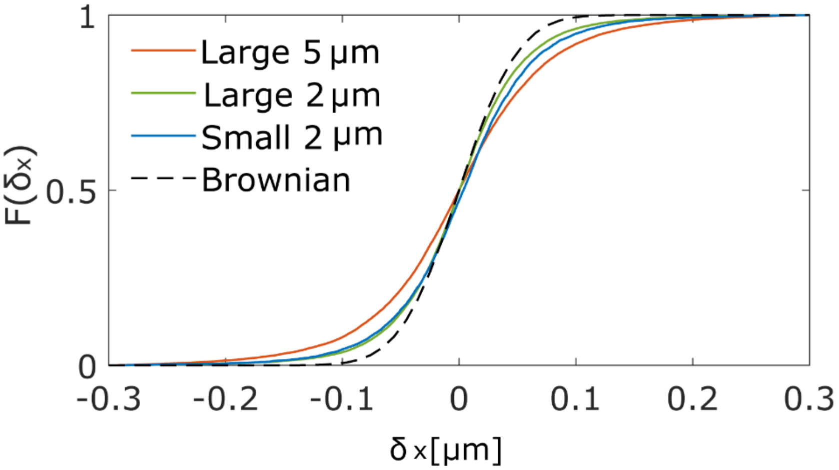

Fig. S4. Cumulative distribution function obtained by KS-test, where aggregates with diameter *d* for columns with pore size 126*d* (orange solid line) and 8d (green solid line), and aggregates with diameter 0.2*d* for the columns with pore size 8*d* (blue solid line).
